# Preliminary Analyses Showed Short-Term Mental Health Improvements after a Single-Day Manager Training

**DOI:** 10.3390/ijerph15010108

**Published:** 2018-01-10

**Authors:** Elena Boysen, Birgitta Schiller, Kathrin Mörtl, Harald Gündel, Michael Hölzer

**Affiliations:** 1 Department of Psychosomatic Medicine and Psychotherapy, University Medical Center Ulm, 89081 Ulm, Germany; harald.guendel@uniklinik-ulm.de; 2 Department of Psychotherapy Science, Sigmund Freud University Vienna, 1020 Vienna, Austria; birgitta.schiller@sfu.ac.at (B.S.); kathrin.moertl@sfu.ac.at (K.M.); 3 Sonnenberg Klinik GgmbH, 70597 Stuttgart, Germany; Michael.Hoelzer@ZFP-zentrum.de

**Keywords:** workplace intervention, common mental disorder, naturalistic design

## Abstract

Psychosocial working conditions attract more and more attention when it comes to mental health in the workplace. Trying to support managers to deal with their own as well as their employees’ psychological risk factors, we conducted a specific manager training. Within this investigation, we wanted to learn about the training’s effects and acceptance. A single-day manager training was provided in a large industrial company in Germany. The participants were asked to fill out questionnaires regarding their own physical and mental health condition as well as their working situation. Questionnaires were distributed at baseline, 3-month, and 12-month follow-up. At this point of time the investigation is still ongoing. The current article focuses on short-term preliminary effects. Analyses only included participants that already completed baseline and three months follow-up. Preliminary results from three-month follow-up survey (*n* = 33, nmale = 30, Mage = 47.5) indicated positive changes in the manager’s mental health condition measured by the Patient Health Questionnaire for depression (PHQ-9: Mt1 = 3.82, Mt2 = 3.15). Training managers about common mental disorders and risk factors at the workplace within a single-day workshop seems to promote positive effects on their own mental health. Especially working with the managers on their own early stress symptoms might have been an important element.

## 1. Introduction

In the past few years, one of the most striking developments in sickness absence is the increase of mental health reasons [1]. The DAK health report states that, in the year 2016, about 17% of the sick leave times in Germany were caused by psychological conditions. Especially common mental disorders (CMD), such as depression and anxiety disorder have been found to put a high risk factor for long-term sickness absence [2].

Trying to explain this increase, psychosocial aspects of the workplace have been examined in several investigations [3]. Therefore, work-related risk factors of CMD can be the working field itself [4], long working hours [5], high job demands and low job control, high work load, low reward, job insecurity [6], and low work social support [7]. Increased levels of stress especially seem to have a great impact on mental health [8].

The workplace, on the other hand, can also function as a resource for mental well-being in terms of social support and good interpersonal relationships [9]. In addition, by spending the days at work, early stress symptoms and behavior changes should be easily detected by colleagues or supervisors and therefore treated rapidly. Rapid intervention proved to be one of the most important factors in the treatment of mental disorders [10].

Knowledge and attitude barriers are the things that hinder those affected and those in contact from approaching and also from seeking help [11]. Quite often, typical prodromal symptoms of mental disorders are not known, so that it is not possible for the supervisor and/or colleague to understand the underlying processes. In addition, there are still stigma and discrimination barriers in the social life concerning mental ill-health. Thus, fears with regard to help offers arise including not knowing where to get help or simply not realizing that help is needed. For employees, this is compounded by fears concerning confidentiality and negative impacts on their career [12].

To meet these underlying circumstances, a wide range of strategies and interventions have been invented in recent years trying to treat and prevent workplace related CMD [13,14,15,16]. Evidence was found for primary prevention interventions and stress management interventions (SMI), based on cognitive behavioral therapy [14,17]. In a meta-analytical approach investigating the efficacy of 55 different SMIs, Richardson and Rothstein [18] found medium-to-large effect sizes across all studies. Especially, interventions based on cognitive behavioral therapy (CBT) elements showed positive effects on mental health parameters. Most of these interventions mainly focus on short-term effects concerning the participants own mental health.

Another important leverage point in dealing with mental health at the workplace is the inclusion of the employees’ supervisors. Montano et al. [17] found, that the leadership style and the leader’s behavior are both related, negatively and positively, to the employee’s mental health and job performance. Workplace health management therefore not only needs the support but the active participation of the company’s managers. An SMI conducted for managers in particular was investigated by Guendel and colleagues in a company in Germany [19]. Modified from a manualized SMI for larger companies by Siegrist and Silberhorn [20], the training mainly focused on three different aims: Improving the awareness of own physical tension, analyzing stress provoking situations using CBT techniques, and teaching of established self-management techniques. A decreased level of perceived stress was found one year after the training. 

In addition to the managers’ own stress management, we assumed that managers that supervise their teams regularly should therefore be able to detect significant changes in their employees’ behavior. They should also know about specific symptoms that indicate mental impairments. Finally, in occurring cases, the managers should feel comfortable and confident enough to seek talks with the salient employee.

Therefore, we implemented a particularly short and distinct intervention for managers in a large company in Germany. Compared to other stress management interventions, we not only addressed the participating managers’ but also their employees’ mental health. By providing basic information on mental ill-health and case study discussions, we wanted to train and sensitize leaders with regard to the mental health condition of their employees.

Within this naturalistic pilot study, we aimed to examine the effects of the manager training. We expected to find improvements in the managers’ knowledge and attitudes concerning mental health. In addition, we wanted to investigate possible positive effects on the particiants’ mental health conditions by focusing on their own stress management for only a short time of the training. In this communication, we want to present some first results from the three-month follow-up of the quantitative survey.

## 2. Materials and Methods

### 2.1. Sample

The present study sampled participants of a manager training in between October 2016 and October 2017. The training was provided at three locations of an industrial company in southern Germany that mainly account for central administration, research, and development as well as purchasing and logistics. Participants were invited by email and word of mouth. At this point of the ongoing intervention, *n* = 71 managers consented to participate whereas three did not provide any contact data and had to be excluded for the follow-up investigation. So far, *n* = 49 already were contacted to fill in the three-month follow-up questionnaires. Data from the remaining participants will be collected within the next months. Out of the contacted participants, *n* = 33 sent back their questionnaires and thus were included in the present analysis.

The managers’ positions ranged from the lowest (team leader) to the second highest (head of department) within the company.

### 2.2. Manager Training

The one-day workshop was provided by the company’s medical and social services as well as by two external experts in groups of 10–15 managers. The training started with one of the company’s staff presenting information about the latest company agreement concerning psychosomatic health at the workplace. This information mainly focused on legal bases and the agreement’s practical application. In the main part of the training, in general an external expert discussed topics about mental illnesses and their relation to the workplace, mainly focusing on depression and burnout syndrome. This part of the training was conducted using various didactic techniques, i.e., initial psychoeducation, interactive lectures, and intensive group discussion. In more detail, in the morning the main focus of the seminar was to enhance the managers´ awareness of their own health. Therefore, a self-awareness exercise about the managers’ own early stress symptoms during recent experiences of more chronic stress was performed and discussed within the group. Based on CBT strategies for situation analysis, the managers were taught to better identify their own thoughts, feelings, and especially their bodily reactions in self-chosen exemplary stress situations. Different possibilities to build up resilience were discussed among participants (‘strengthening one´s own health’). After lunch, the main focus was to care for and to address issues concerning the health of employees working with the managers. Thus the external expert asked the managers to share real experiences of current difficult situations they experience with their employees concerning chronic stress related symptoms or even beginning mental illness. Real such cases were discussed in depth within the group. Professional information about how to deal with employees displaying initial behavioral problems (‘paying attention to health of employees’) was added in between the group discussion. The external expert especially focused on improving communication skills based on a more psychotherapeutic approach (e.g., active listening, structuring a conversation, or how to deal with emotions; role play). At the end of the day, one of the industrial council members gave further information about the company’s help offers.

### 2.3. Quantitative Instruments

The questionnaire combined several valid instruments with single item questions in two main parts: The first half of the questionnaire focused on the participants’ knowledge and attitudes concerning mental health. The second part focused on the participants’ own health and their working situation. Additionally, socio-demographic and personal contact data were collected at the baseline assessment. As this short communication only focuses on health changes, the first part of the questionnaire will be reported elsewhere.

Physical and mental health was measured by the 12-Item Short Form Health Survey (SF-12, [21]) and the 9-Item Depression Scale of the Patient Health Questionnaire (PHQ-9, [22]). Using single-items, the SF-12 enquires as to the respondent’s subjective perception of his or her own daily functioning (e.g., “In the past 4 weeks, did you experience any difficulties at work or at any other daily activity according to your physical health?”) [21]. Item scores are summed up to the two scales physical health and mental health (range 0–100). The PHQ-D measures the self-reported occurrence of depressive symptoms by difficulties in the preceding two weeks (e.g., “fatigue and the feeling of a loss in energy”) [22]. Scoring (sum scores ranging 0–40) is conducted according to the diagnostic criteria of the DSM-IV. Thereby, a cut-off score of 10 indicates a need for treatment.

To learn more about the managers’ working situation, the Effort–Reward Imbalance Inventory (ERI, [23]) and the Irritation Scale (IS, [24]) were included in the survey. The ERI assesses psychosocial working loads on three scales: (1) The required cost, that needs to be invested in the daily work (effort, range 6–26); (2) the possible benefits that come along with the work, such as financial or interpersonal gains (reward, range 10–40); and (3) the tendency to consider work too much, also in the time off (overcommitment, range 4–24) [23]. The sum score of the effort and the reward scale can be set into relation. The IS measures the subjectively perceived emotional and cognitive strain related to the employment [24]. Therefore, eight items asses the employee’s ability to relax and regenerate after work (e.g., “even at home I think about working issues“). Single items are summed up to one global score (range 1 to 7).

Some further questions were added. These single items aimed at how the managers feel about their own mental health (“What do you think about your own mental health condition?”), how they would feel about being affected by a mental disorder (e.g., “Would you feel ashamed about having a mental disorder?”) and what they think about their company’s offers regarding mental health (e.g., “Are colleagues affected by a mental disorder being supported and treated fairly by colleagues and supervisors?”). Answers could be given on nine-point Likert scales. At the end of the questionnaire, participants were asked whether they would like to receive a feedback on their personal health questionnaires (SF-12, PHQ). Feedback was given according to sex and age specific norm samples. Scores were differentiated into five categories according to standard deviation (SD): more than one (two) SD lower than the average, in between the average and more than one (two) SD higher than the average.

### 2.4. Qualitative Instruments

To gain further information about the implementation process of the manager training, semi-structured interviews were conducted additionally. Interviews were planned at baseline and 12-month follow-up and took about 30 min each. Managers were asked about their reasons to participate in the training and how they think about the trainings’ necessity. Also, they were invited to talk about the changes they experienced since the implemenation of the trainings started. 

At this point of the investigation, the accompanying interviews are not completely analyzed yet. A detailed evaluation of the qualitative data will be reported elsewhere.

### 2.5. Implementation

Information about the training offer was provided on the company’s intranet. As one of several further education offers, managers voluntarily signed in for the single-day workshop. All trainings were implemented in seminar rooms located at the company’s site. Rooms offered sufficient capacity for the group size and were equipped with a computer and projector.

A few days in advance, participants were informed about the associated study via email including the questionnaire. On the workshop’s day, the trainer informed the managers again about the study and handed out a detailed participants’ information, a form to give written consent, the questionnaire, and a reply-paid envelope. The participants were then given 10 min before the training started to fill in the first half of the questionnaire. Afterwards they were told to answer the rest of the questions later and send it back to the clinic, if they were interested to participate in the study.

Three months after the training, all study participants received another package by mail including the follow-up questionnaire and a reply-paid envelope. They were asked to the questions and send the questionnaire back to the clinic. On a voluntary basis, participants received feedback regarding their health-related questionnaires via mail.

### 2.6. Design and Data Analysis

The present study was conducted in a naturalistic design. The change of mental health measures (baseline to follow-up) was analyzed using paired sample *t*-tests (two tailed) with an alpha set to 0.05. Effect sizes were calculated for repeated measures [25]. All data management and statistical analyses were conducted using IBM SPSS statistics 24 (IBM Corporation, Armonk, NY, USA).

### 2.7. Ethical Approval and Registration

The study was conducted in accordance with the Declaration of Helsinki, and the protocol was approved by the Ethics Committee of the Ulm University (326/16). The investigation has been registered by the German Clinical Trials Register (DRKS) under the identification DRKS00011371.

## 3. Results

### 3.1. Descriptives

Socio-demographic and employment information at baseline are presented in Table 1. No significant differences were found between the analysis sample (participants completed baseline and follow-up) and the incomplete sample. All received questionnaires were filled in completely, except for a very few missing single items. Missing data was handled according to each authors’ instructions.

### 3.2. Physical and Mental Health

A two-tailed paired samples *t*-test showed significant improvements in depressive symptoms measured by the PHQ (t (32) = 2.72, *p* = 0.010). The effect size of the difference was d_Repeated measures_ = −0.275. Also, a single item of self-reported psychological health showed a significant improvement in between baseline and follow-up with t (32) = 3.20, *p* = 0.003.

There were no significant changes at the physical and mental health scales of the SF-12. Table 2 outlines the data in detail.

## 4. Discussion

Within this investigation, we wanted to learn about the preliminary effects of a single-day manager training addressing own as well as employees’ mental health at the workplace. As a first result after a time period of three months, managers reported improved mental health conditions. Educating managers in dealing with their own mental health seems to have lead to a better approach to their own individual stress factors and therefore lower depressiveness.

A controlled long-term investigation on the efficacy of a stress-management intervention program for managers showed long-lasting effects on the participants’ mental health over a time period of nine years [26]. The program was based on a psychodynamic approach combined with CBT elements such as psychoeducation, working on cognitions and individual resources. The training was conducted over two consecutive days, only focusing on the participants’ own stress management. As for the single-day training investigated in the current communication, the focus on the managers’ own mental health only took a few hours, but yet improved mental health was reported in this short-term pilot investigation. Given the fact that this study was conducted in a non-randomized and uncontrolled design, it is not possible to clearly ascribe these improvements to the training. There are other variables we did not measure that could have influenced the findings. Still, these early results might imply that even short stress management interventions may improve managers’ handling with their individual sources of stress. The quantitative instruments used and analyzed in this investigation seemed to be well received by the participants and thus helped to facilitate a first, interesting gain of knowledge. They also presumably captured a scope that shows real changes.

Not only a better handling, but an increased awareness of the own mental well-being could have had a positive effect on mental health. In the main study, we also collected qualitative data, to learn more about changes going on in the company. Therefore, we interviewed some of the managers participating in the training. By asking the participants about who they think could be in need of psychological support at some time, most of the interviewees agreed it could be anyone: “[...] it does not only concern employees but supervisors as well”. This might support the suggestion about the managers becoming more aware of themselves likewise. Giving personalized feedback to the managers’ health-related questionnaires could have intensified this effect. 

Also, an increased knowledge and therefore higher confidence in dealing with the topic could have had further positive influences on the participant’s mental health. Dimoff, Kelloway, and Burnstein [27] found positive effects on self-efficacy related to employee mental health after offering three hours of mental health awareness training. Another investigation showed higher levels of knowledge of the managers’ role and confidence in communicating with employees after visiting a workplace mental health training [28]. Asking the managers about their confidence in dealing with their employees, they reported about feeling more secure: “The training helped to have a first lead for conversations [with employees that show behavior changes]” or “As a manager, it is important to know that there are places to go [to get help within the company]”.

This pilot study showed first indications for the efficacy of a short intervention regarding mental health at the workplace. To examine the effects of a single-day training addressing the participants’ and their employees’ mental health conditions, further research is needed in a randomized controlled setting. In addition, long-term investigations will clarify the sustainability of a short intervention or whether a higher dose is needed to gain persistent effects.

Focusing on knowledge and attitude changes regarding the training provided, results from a 12 months follow-up will be reported elsewhere.

### Limitations

A limitation of these results is the data selection procedure. Data collection is still in progress, so we only included participants that completed both the baseline and follow-up questionnaires. Therefore, we have no further information about whether the remaining participants dropped out the study, skipped the three-month follow-up or just had not replied yet.

## 5. Conclusions

This pilot investigation showed a first positive direction for the short-term effectiveness of a single-day manager training regarding mental health. Compared to other stress management interventions, this training not only focused on the participants but their employees’ mental health condition as well. Although improvement of mental health parameters has been reported after three months, further research is needed to clarify the efficacy and its stability over a longer period of time.

## Figures and Tables

**Table 1:** Demographic and employment information at baseline for all included participants so far and for a selected group that already gave information at a three-month follow-up time point

	Baseline and Three Months Follow-Up (*n* = 33)	Baseline and No Follow-Up Yet (*n* = 38)	All Included Participants So Far (*n* = 71)
Male gender *n* (%)	30 (90.9)	27 (81.8)	61 (85.9)
Age at baseline M (SD)	47.5 (8.8)	47.0 (7.9)	47.2 (8.5)
In partnership *n* (%)	30 (90.9)	29 (87.9)	64 (90.1)
People in household M (SD)	3.4 (1.2)	3.6 (1.2)	3.5 (1.2)
Position **^1^**			
Stage A *n* (%)	4 (12.1)	2 (6.1)	7 (9.9)
Stage B *n* (%)	15 (45.5)	14 (42.4)	29 (40.8)
Stage C *n* (%)	8 (24.2)	7 (21.2)	18 (25.4)
Stage D *n* (%)	1 (3.0)	4 (12.1)	5 (7.0)
Others **^2^** *n* (%)	5 (15.2)	5 (15.2)	11 (15.5)
Personnel responsibility M (SD)	12.7 (10.5)	54.2 (183.6)	34.3 (123.5)
Working hours/week M (SD)	45.8 (6.1)	43.5 (12.5)	45.0 (9.7)

**^1^** Position in the company ranging from stage A (lowest) to stage D (highest). **^2^** Managers without a classifiable position, e.g., top management.

**Table 2:** Mean score differences of the measured scales in between baseline and three months follow-up.

*n* = 33	M_Baseline_ (SD)	M_Follow-up_ (SD)	d_Repeated measures_
SF-12 **^1^** physical health	53.08 (6.73)	52.37 (4.91)	−0.11
SF-12 mental health	49.17 (9.49)	51.35 (9.07)	0.31
PHQ-9 **^2^** depression	3.82 (2.53) **	3.15 (2.56) **	−0.28
MH **^3^** self-reported	2.73 (1.35) **	2.35 (1.14) **	−0.54

**^1^** 12-Item Short Form Health Survey [21]—higher scores stand for better health conditions; **^2^** Patient Health Questionnaire [22]; **^3^** self-reported mental health, ** significant at 0.01 level.
